# Overexpression of a Pak Choi Gene, *BcAS2*, Causes Leaf Curvature in *Arabidopsis thaliana*

**DOI:** 10.3390/genes12010102

**Published:** 2021-01-15

**Authors:** Ye Lin, Hualan Hou, Yuhang Zhang, Xilin Hou

**Affiliations:** 1 State Key Laboratory of Crop Genetics and Germplasm Enhancement, Nanjing Agricutural University, Nanjing 210095, China; 2018104050@stu.njau.edu.cn (Y.L.); 2017204021@njau.edu.cn (H.H.); 2018104049@njau.edu.cn (Y.Z.); 2 Key Laboratory of Biology and Germplasm Enhancement of Horticultural Crops in East China, Ministry of Agriculture, College of Horticulture of Nanjing Agricultural University, Nanjing 210095, China; 3 Engineering Reserch Center of Germplasm Enhancement and Utilization of Horticultural Crops, Ministey of Education, Nanjing Agricutural University, Nanjing 210095, China

**Keywords:** *BcAS2*, *BcAS1*, leaf polarity, pak choi, transgenic

## Abstract

The LBD (Lateral Organ Boundaries Domain) family are a new group of plant-specific genes, which encode a class of transcription factors containing conserved Lateral Organization Boundary (LOB) domains, and play an important role in regulating the adaxial–abaxial polarity of plant leaves. In *Arabidopsis thaliana*, ASYMMETRIC LEAVES 2 (*AS2*) has a typical LOB domain and is involved in determining the adaxial cell fate. In this study, we isolated the *BcAS2* gene from the pak choi cultivar “NHCC001”, and analyzed its expression pattern. The results showed that the *BcAS2* encoded a protein made up of 202 amino acid residues which were located in the nucleus and cytomembrane. The Yeast two-hybrid system (Y2H) assay indicated that *BcAS2* interacts with *BcAS1-1* and *BcAS1-2* (the homologous genes of *AS1* gene in pak choi). In the transgenic *Arabidopsis thaliana* that overexpressed *BcAS2* gene, it presented an abnormal phenotype with a curly shape. Taken together, our findings not only validate the function of *BcAS2* in leaf development in *Arabidopsis thaliana*, but also contribute in unravelling the molecular regulatory mechanism of *BcAS2*, which fulfills a special role by forming complexes with *BcAS1-1/2* in the establishment of the adaxial–abaxial polarity of the lateral organs in pak choi.

## 1. Introduction

The flat symmetric leaves are essential for plant development as they provide the place for photosynthesis, respiration and other important physiological activities. It is reported that the leaves originate from the shoot apical meristem (SAM) along proximal–disal, adaxial–abaxial and medial–lateral axis [1,2]. Therefore, the successful establishment of SAM is crucial for the leave polarity [3,4]. The initiation and maintenance of SAM requires the normal expression of a highly conserved class of homeodomain transcription factors encoded by class-I KNOX genes, which include four members: SHOOT MERISTEMLESS (*STM*); BREVIPEDICELLUS (*BP*); *KNAT2* and *KNAT6* [5,6,7,8]. The abnormal embryos lacking SAM appear in the loss-of-function mutation in *STM*, which also fail to develop any postembryonic vegetative tissue [9,10]. However, the development of leaves will be disrupted if the KNOX genes continue expressing. Hareven’s study demonstrates that there would be super-compound leaves if the KNOX genes overexpressed in tomato [11]. The ectopic expression of KNOX genes in maize presents an abnormal phenotype with distal displacement of sheath and auricle tissue into the blade, and produces deeply serrated or segmented leaves in *Arabidopsis thaliana* [12,13]. Therefore, it is a critical event to down regulate KNOX genes in lateral organ primordia for normal leaf development [14].

ASYMMETRIC LEAVES 2 (*AS2*), containing a Lateral Organization Boundary (LOB) domain, is a key determinant of adaxial cell fate. It is involved in the leaf morphogenesis and downregulates the expression of KNOX genes via forming a complex with an MYB family transcription factor (TF) *AS1* [15,16]. From a previous study, some other components also cooperate with the AS2-AS1 complex in order to maintain the normal development of leaves, such as several TCP family proteins, namely TEOSINTE BRANCHED1, CYCLOIDEA, and PCF (*TCP*) [17]. In addition, some epigenetic factors also participate in the pathway of KNOX gene silencing as a partner with AS2-AS1, for instance, HISTONE REPRESSION A FACTOR (HIRA) [18], HISTON DEACETYLASE HDA6 [19]. The AS2-AS1 complex also restrict the YABBY, ARF, and KANAND family genes, which play important roles in deciding adaxial–abaxial polarity during leaf development [20,21]. These results indicate that AS2-AS1 is essential in the establishment of plant leaf polarity. 

The interaction mechanism between *AS2* and *AS1* has been studied by many researchers in *Arabidopsis thaliana*. We know that *AS2* can interact with *AS1* in vitro using a yeast two-hybrid system assay [22], and *AS2*-*RS2*(the homologous gene of *AS1* in maize) are co-located in the nucleus [23,24]. The latest research demonstrates that the *AS2*, with the zinc-finger DNA-binding motif, is indispensable for the establishment of perinuclear bodies which influences the normal development of leaves [25]. The transgenic lines that overexpress the *AS2* gene using the 35S promoter in cockscomb present significantly curly leaves [26].

Pak choi (*Brassica rapa* ssp. *chinensis*), belongs to the *Brassica* genus of Cruciferae and has a close relationship with *Arabidopsis thaliana*. As a relatively important leafy vegetable, it is popular and widely cultivated in Asia. The leaf morphology is a vital agronomic trait with a great impact on pak choi architecture and yield, while the establishment of leaf polarity is a key event in its leaf development. From a previous study, we know that the *AS2* gene is indispensable for adaxial patterning of lateral organs. However, the function of *AS2* in pak choi is still unknown. In the present study, we firstly isolated *BcAS2* from the cultivar of the pak choi (NHCC001) in order to discover its regulatory mechanism in lamina development. We demonstrated that *BcAS2* physically interact with the *BcAS1-1/2* in vitro and in vivo via a Y2H assay and colocalization assay. Furthermore, we successfully obtain the transgenic lines overexpressed *BcAS2* with an obvious leaf curly phenotype. These findings extend our understanding of *AS2* in Cruciferae plants, suggesting that *BcAS2* and *BcAS1-1/2* were involved in the establishment of leaf polarity in the form of complex in pak choi.

## 2. Materials and Methods 

### 2.1. Plant and Growth Conditions

The seeds of pak choi cultivar “NHCC001” were provided by Professor Xilin Hou (Nanjing Agricultural University) and grown in illumination incubators under the conditions of light 16 h/24 °C and dark 8 h/24 °C, subsequently. The different tissue (leaf, lobus cardiacus, stem, root, hypocotyl) of 1 month old seedings were sampled and frozen immediately in liquid nitrogen and stored at −70 °C. *Nicotiana benthamiana* and *Arabidopsis thaliana* wild type (WT) used in this study were grown in illumination incubators under the same conditions.

### 2.2. Sequence and Analysis of BcAS2

We designed primers based on the sequence of Bra039733 (http://brassicadb.org/brad/) to clone the full-length CDS (coding sequence) of *BcAS2* from a pak choi template by homology cloning according to our previous report (Table S1) [27,28]. The physicochemical characteristics of BcAS2 protein were analyzed by the Expasy website (https://web.expasy.org/protparam/), hereinafter referred to as Protparam. The homologous genes of *BcAS2* were searched by the online BLAST sever (https://blast.ncbi.nlm.nih.gov/Blast.cgi). The multiple sequence alignments of homologous proteins were performed through DNAman (download from https://www.lynnon.com/dnaman.html). The conserved motifs were analyzed using the MEME website (http://meme-suite.org/tools/meme). The potential proteins interacting with BcAS2 were predicted by online STRING software (https://version11.string-db.org/cgi/network.pl?taskId=xScSvWWALyMg). We downloaded the protein sequences of predicted results from the Brad website (http://brassicadb.org/brad/searchGene.php) and traced the homologous genes in *Arabidopsis thaliana* through the blast tool on the Tair website (https://www.arabidopsis.org/Blast/index.jsp).

### 2.3. Y2H Analysis

The full-lengths of *BcAS2*, *BcAS2-1*, *BcAS2-2*, *BcAS1-1* and *BcAS1-2* were amplified by PCR using PrimerSTAR Max Premix (TaKaRa, Dalian, China) from the cDNA of “NHCC001”. After DH5α transformation (TOLOBO, Shanghai, China), they were successfully cloned into pGADT7(AD) and pGBKT7(BD). We cut *BcAS2* for *BcAS2-1* (containing LOB domain) and *BcAS2-2* for transcript activation ability analysis. The pGBKT7-*BcAS2/BcAS2-1/BcAS2-2* and pGADT7 were co-transformed into the yeast strain Y2H Gold. We estimated the transcript activation ability by observing the growth of transformants on the selected medium lacking leucine and tryptophan (SD/-Leu/-Trp) and selected medium lacking leucine, tryptophan, adenine, and histidine (SD/-Leu/-Trp/-Ade/-His) after 3–4 days. The interaction between them was tested using the same way.

### 2.4. Subcellular Localization

The protein-coding region of *BcAS2* was cloned into pENTRTM D-TOPO (Invitrogen, Carlsbad, CA, USA) firstly, then the 35S: *BcAS2*-GFP was generated by LR reaction between pENTRTM D-TOPO-*BcAS2* and pEarlyGate103 vector for subcellular localization. The *BcAS2*, *BcAS1-1/2* were cloned into pCAMBIA1302-mcherry and pCAMBIA1302 respectively to generate the *BcAS2*-mcherry and *BcAS1-1/2*-GFP fusions for co-localization. The main primers used in our study are listed in Table S1. These plasmids were transformed into *Agrobacterium* strain GV3101 for infiltrating into tobacco leaves. Then the leaves were co-injected with 35S:*BcAS2*-GFP/35S: GFP and DAPI (nucleus specific dye). About 70 h after infiltration, we collected the images by confocal laser scanning microscopy (Zeiss, LSM 500, Oberkochen, Germany).

### 2.5. Agrobacterium-Mediated Transformation

To obtain transgenic plants overexpressing *BcAS2*, *Arabidopsis thalina* (Columbia) was transformed with 35S:pEarleyGate103-*BcAS2*-GFP using the floral-dip method [29]. The T0 seeds of the transgenic plants were screened on MS solid medium containing 50 mg/L Basta and 16 mg/L Timetin. After about 10 days, only the positive seedlings could grow normally. The seeds obtained from the positive seedlings were transgenic seeds of the T1 generation, which were further selected on MS solid medium with the same resistance until T3 generation. Then the T3 lines were selected for the qPCR analysis and phenotypic observation.

### 2.6. Real-Time Quantitative PCR (qPCR) Analysis

The total RNA was extracted by RNA simple total RNA extraction kit (TIANGEN Beijing, China). The cDNA was synthesized using the PrimeScript TM II 1st Strand cDNA Synthesis Kit (TaKaRa, Dalian, China). The *AtActin* of *Arabidopsis thaliana* and *BcActin* of pak choi were used as house-keeping genes, respectively. Gene-specific primers used in this study are given in Table S1. The qPCR was performed using SYBR Green Master Mix (Yeasen, Shanghai, China). The data were calculated using the 2^−ΔΔCT^ method [30].

## 3. Results

### 3.1. Sequence and Expression Analysis of BcAS2 

**Figure 1:**
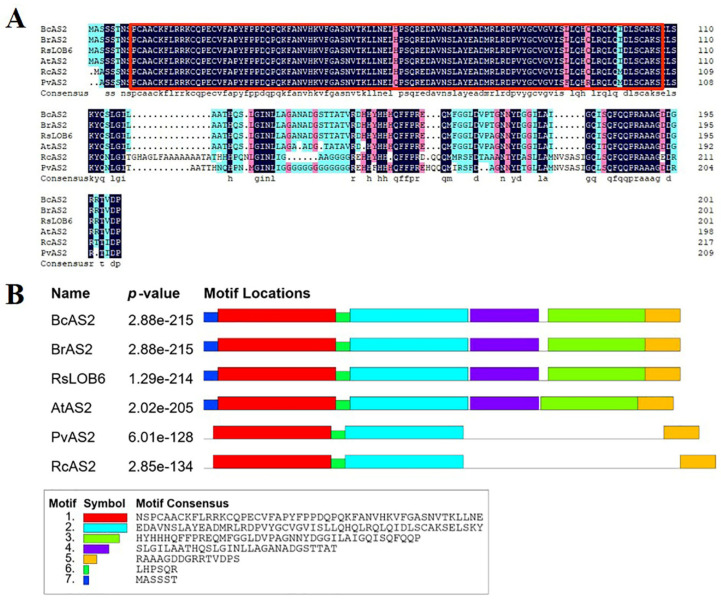
The sequence analysis of BcAS2 protein. (**A**) The multiple sequence alignment of BcAS2 protein with AS2-like proteins in *Brassica rapa (BrAS2,* XP_009127444.1*)*, *Raphanus sativus (RsLOB6*, XP_018468477.1*)*, *Arabidopsis thaliana* (*AtAS2,* NP_001077777.1), *Pistacia vera (PvAS2,* XP_031287864.1*)*, *Ricinus communis* (*RsAS2,* XP_002514766.1). The red box contained the LOB conserved domain in different species. (**B**) The motif analysis of BcAS2, the relevant sequence information is shown at the bottom of the figure, the *p*-value represents the significance of each motif.

The online software ProtParam showed that the full length coding sequence (CDS) of *BcAS2* encoded 202 amino acid residues with molecular weight of 22.09 kDa and theoretical isoelectric point (pI) of 7.1. Multiple sequence alignment of BcAS2 and AS2-like proteins from different crops (*Brassica rapa, Raphanus sativus, Arabidopsis thaliana, Pistacia vera, Ricinus communis*) showed that they had a highly conserved LOB domain at the N-temial(9-107 amino acid sites) (Figure 1A). In addition, the results of motif analysis indicated that there were identical motifs among BcAS2, BrAS2 and RsLOB6 proteins which were similar to motifs in AtAS2. Interestingly, they all belong to Cruciferae, suggesting that the function of *BcAS2* gene in pak choi may be similar to that in *Arabidopsis thaliana* (Figure 1B). 

**Figure 2:**
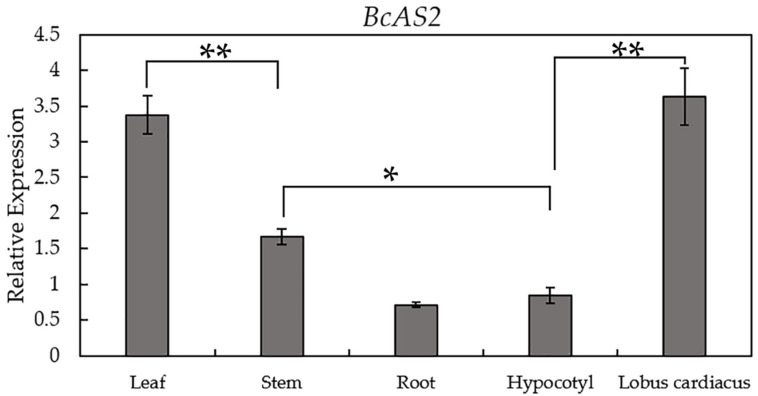
Expression patterns of *BcAS2* in different tissues. The expression level of *BcAS2* in leaf, stem, root, hypocotyl, lobus cardiacus of 1-month-old pak choi cultivar “NHCC001”. The data represent the average of the three replicates, and the error bars represent the standard deviation between the replicates. ** and * mean significant differences between different tissues, *, *p* < 0.05, **, *p* < 0.01.

To verify the expression pattern of *BcAS2* in different tissues of pak choi, the qPCR assays were performed to identify the expression level using the samples of 1-month old “NHCC001”. The results showed that *BcAS2* was highly expressed in the lobus cardiacus and the leaves, followed by stems, but lower in roots and hypocotyls (Figure 2). This result was consistent with the previous studies on the role of *AS2* gene in leaf development [16,23].

### 3.2. Subcellular Localization of BcAS2 Protein

**Figure 3:**
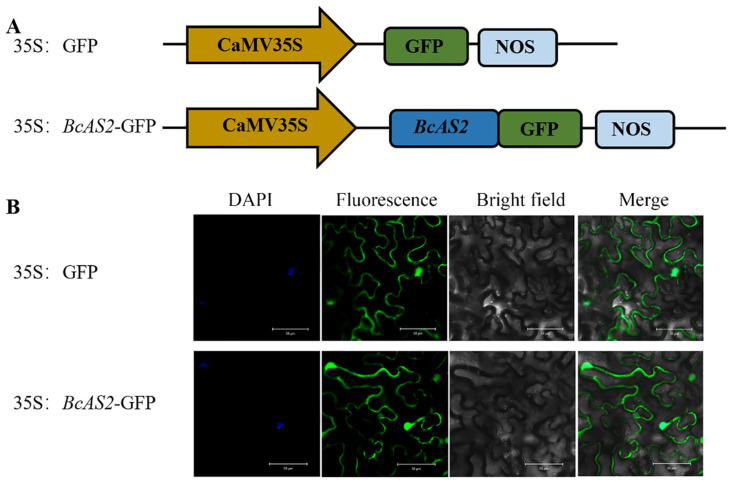
Subcellular localization of *BcAS2*. (**A**) The construct of 35S: GFP and 35S:*BcAS2*-GFP. GFP: green fluorescent protein; NOS: nopaline synthase gene. (**B**) Transient expression of 35S: GFP and 35S:*BcAS2*-GFP fusion protein with DAPI (nucleus specific dye) in tobacco. Scale bars = 50 μM.

To investigate where *BcAS2* functions in the leaf cell, we observed the localization of *BcAS2* using the *Agrobacterium* injection method. From the images captured, we found that the protein of 35S:*BcAS2*-GFP fusions were expressed in the nucleus and cytomembrane mainly (Figure 3), indicating that *BcAS2* may not only function as a transcription factor but also as some cytomembrane proteins.

### 3.3. Interaction Between BcAS2 and BsAS1-1/2 In Vivo and In Vitro

In total, 10 potential interacting proteins of BcAS2 were predicted by online software STRING. Based on the blast result of the Tair database, we found that these proteins mainly belonged to four gene families, including KANADI family (Bra023570, Bra008613, Bra033844, Bra023254), YABBY family (Bra037320, Bra00538), MYB family (Bra000011, Bra005177), and KNOX family (Bra000638, STM) (Figure S1). The result of multiple sequence analysis showed that the proteins identified in Figure S1 were highly homologous to *AtKAN1, AtKAN2, AtYAB2*, and *AtYAB3* (Figure S2). Among these results, we were surprised to find that AS1 is one of the proteins that might interact with BcAS2, for many researches in *Arabidopsis thaliana* have demonstrated that *AS2* and *AS1* usually function as a complex [22,24]. 

**Figure 4:**
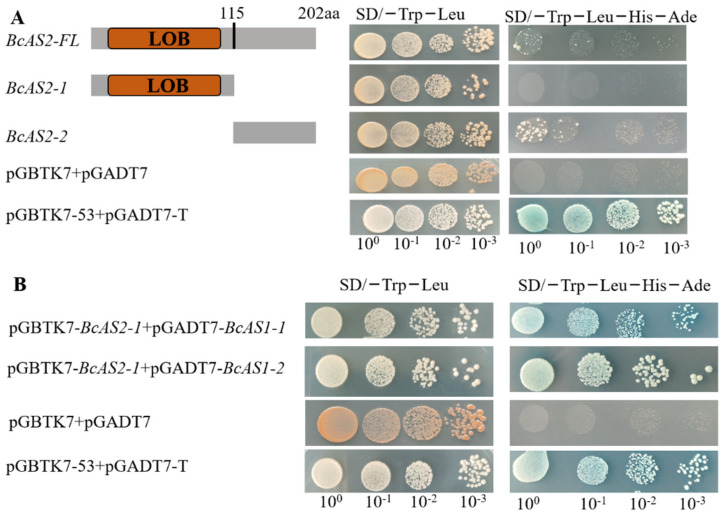
Validation of interaction between BcAS2 and BcAS1-1/2 in vivo. (**A**) Transactivation activin analysis of *BcAS2*. *BcAS2*-FL, *BcAS2*-1 and *BcAS2*-2 represented the full-length, the region containing Lateral Organization Boundary (LOB) domains and the rest region of BcAS2, respectively, which were cloned into pGBTK7 (BD) and co-transformed into Y2H gold strains with pGADT7 (AD). (**B**) Interaction validation of *BcAS1*-1/2 (two homologous genes of *AS1* gene in pak choi) and *BcAS2*, pGADT7 + pGBKT7 and pGADT7-T + pGBKT7-53 were used as the negative and positive control.

From a previous study, we realized that the AS2 of *Arabidopsis thaliana* had no transcript activation ability [12], but it still needed further verification in pak choi. Therefore, the yeast two-hybrid screen method was performed in our study to validate our hypothesis. The result showed that the yeast Y2H gold strains co-transformed with pGBTK7-*BcAS2*/*BcAS2-2* and pGADT7 (empty vector) could grow well on SD/-Trp/-Leu/-His/-Ade medium as well as the positive control (pGBTK7-53 + pGADT7-T). While the *BcAS2-1* and pGADT7 failed to grow on SD/-Trp/-Leu/-His/-Ade medium, indicating that the full length of *BcAS2* had transcript activation ability while *BcAS2-1* had not (Figure 4A). In order to further verify the interaction between *BcAS2* and *BcAS1-1/2*, we generated the yeast Y2H gold strains co-transformed with pGBTK7-*BcAS2-1* and pGADT7-*BcAS1-1/2*. The result emerged that all the experimental groups can grow on selective medium (SD/-Trp/-Leu/-His/-Ade), while the negative control cannot grow, illustrating that the BcAS2 protein interacted with BcAS1-1 and BcAS1-2 proteins (Figure 4B).

**Figure 5:**
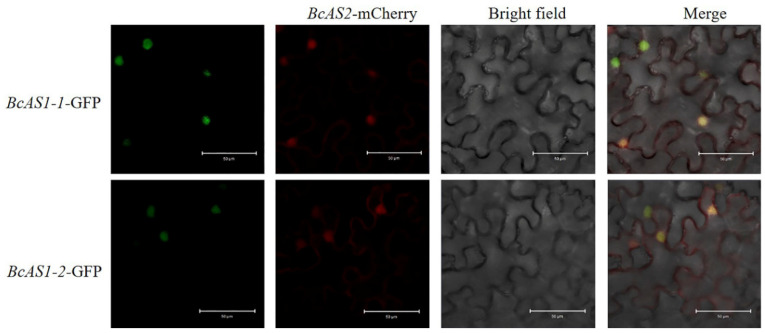
The *BcAS2*-mCherry and *BcAS1-1/2*-GFP were co-localized in the nucleus. From left to right, the fluorescence signals in the nucleus, nucleus, and cytomembrane, nucleus due to *BcAS1-1/2-*GFP, *BcAS2-*mChery and the co-expression between them, respectively. Scale bars = 50 µM.

Based on the interaction between the *BcAS2* and *BcAS1-1/2* in the Y2H system, we further confirmed their relationship by a co-localization assay in tobacco leaves transiently co-infiltrated with the *BcAS2*-mcherry and *BcAS1-1/2*-GFP. From the images we captured, we can observe that the red fluorescence of *BcAS2*-mcherry fusion protein was mainly expressed in the cell membrane and nucleus, which is also consistent with the subcellular localization result of *BcAS2* in this study. The green fluorescence of *BcAS1-1/2*–GFP was mainly expressed in the nucleus. The co-expression of *BcAS2* and *BcAS1-1/2* in the nucleus resulted in fusion yellow fluorescence (Figure 5). The interaction between *BcAS2* and *BcAS1-1/2* in the yeast two-hybrid system and the co-localization between them allowed us to propose a mechanism that they might also form a complex to function as a leaf polarity regulator in pak choi.

### 3.4. Overexpression of BcAS2 Resulted in Upward-Curling Leaves and Downregulated Some Polarity-Related Genes

**Figure 6:**
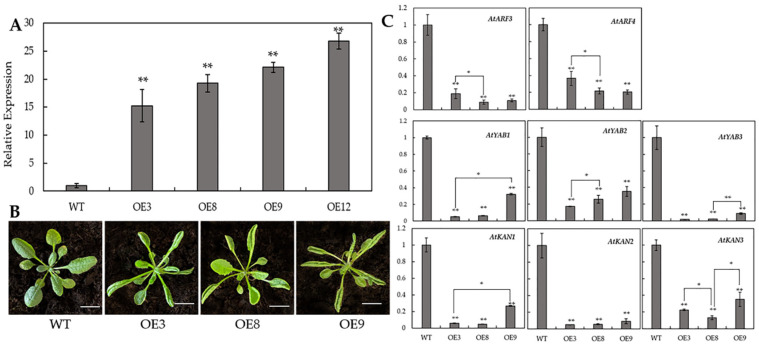
The analysis of transgenic *BcAS2* lines. (**A**) Expression levels of *BcAS2* in four positive overexpression (OE) lines and wild type (WT), respectively. ** means significant differences compared to WT (*p* < 0.01). (**B**) Phenotype of the OE *BcAS2* lines. Scale bar = 5 cM (**C**) The expression level analysis of eight polarity-related genes have been reported in *Arabidopsis*. The value of genes expression level in WT was set to ‘1’ as a control. The data represent the average of the three replicates, and the error bars represent the standard deviation between the replicates, *, *p* < 0.05, **, *p* < 0.01.

To understand better the regulation of *BcAS2* during leaf polarity formation in Pak choi, we generated the transgenic *Arabidopsis thaliana* with the ectopic expression in *BcAS2*. The result of qPCR reveled a much higher transcript level of *BcAS2* in overexpression lines (named as OE3, OE8, OE9, OE12) compared with WT (Figure 6A). Further, it was obvious that the leaves of overexpression lines presented the upward curling phenotype (Figure 6B), which is in line with the transgenic *AS2* lines in *Celosia cristata* [26]. We further examined the transcription levels of eight genes (*AtYAB3*, *AtYAB2*, *AtYAB3*, *AtKAN1*, *AtKAN2*, *AtKAN3*, *AtARF3*, *AtARF4*) which have been reported that they were related to leaf abaxialization in a previous study [31,32]. Our result showed that the transcript levels of the eight genes were extremely reduced in transgenic *BcAS2* lines compared with WT (Figure 6C). Therefore, we speculated that the curly leaves of *BcAS2* transgenic plants may be attributed to the interaction between abaxial and abaxial polarity regulators, but the specific regulatory pathway remains to be studied.

## 4. Discussion

The leaves play an important role in the success of plants which are regulated by a complex genetic network [33]. The polarity of the plant’s lateral organ is established and young leaves are formed with the continuous division of leaf cells among the three asymmetrical axes. Bowman found that only when the adaxial-abaxial polarity was established can the leaf dysplasia begin [34]. Among these leaf polarity regulators, the LBD gene family encoded a class of protein with an LOB domain only found in plants, which regulates the plant-specific processes in many species. While the biological role of LBD genes in pak choi is poorly understood. Here, we isolated the *BcAS2* gene from pak choi cultivar “NHCC001”, which had a typical LOB domain and was homologous to *AS2* in *Arabidopsis*. The multiple sequence and motif analysis revealed a high similarity between *BcAS2* and *AS2*, implying that *BcAS2* may also participate in specifying the latter’s organ shape in pak choi. We profiled the expression level of *BcAS2* in different tissues at the 1-month-old stage. From the results, we found that the expression of *BcAS2* was higher in leaves and the lobus cardiacus compared with other tissues, which allowed us to speculate that *BcAS2* mainly played a role in the leaves of pak choi.

Previous reports showed that *AS2* mediated the adaxial polarity with a MYB TF *AS1* as a complex [33]. Therefore, we want to verify whether there is a similar mechanism in the regulation of *BcAS2* on leaf development in pak choi. We predicted the potential proteins that might interact with BcAS2 using the online server STRING (Figure S1). From the results, we obtained 10 potential proteins, including BcAS1-1/2. To gain further insight into their relationship, we performed a yeast two-hybrid screen to verify our conjecture. As expected, the result revealed that BcAS2 could bind with BcAS1-1/2 in yeast strains. In order to explain the interaction between them in more depth, we utilized a co-localization assay to analyze the localization under the *Agrobacterium tumefaciens*-mediated transient transformation system. According to the picture we captured, it was evident that they co-expressed in the nucleus, which was in line with a previous study [35].

We first overexpressed *BcAS2* in *Arabidopsis thaliana* to investigate the function of *BcAS2* more intuitively. Through phenotypic observation, we found that the over-expressed *BcAS2* phenotypes of transgenic lines reflected dramatically curved leaves, which were similar to kan1 and kan2, and arf3 and arf4 double mutants but different from the transgenic *Arabidopsis thaliana* with a high expression in a wheat YAABY gene, *TaYAB1* [36,37,38]. Considering that the homologous genes of *BcAS2* potential interaction genes in *Arbidopsis thaliana* and the main function of KANADI ARF and YABBY genes are promoting the development of leaf abaxial axis [39,40,41,42], we speculated that the high transcript level of *BcAS2* in the plant results in the decreased expression of some abaxial polarity relative genes to improve the adaxial development. With this in mind, we examined eight genes that are vital in the determination of abaxial cell fate, namely *AtKAN1*, *AtKAN2*, *AtKAN3*, *AtYAB1*, *AtYAB2*, *AtYAB3*, *AtARF3*, and *AtARF4* [34,35,36]. The result indicated that they were evidently down-regulated in transgenic lines compared with WT, especially the *AtKAN1, AtKAN2*, *AtARF3*, and *AtYAB3*, which was positive for our conjecture. Significantly, the elevated *BcAS2* expression level also downregulated the *AtARF3* and *AtARF4*, which responded to auxin, an important endogenous hormone participating in the regulation of plant development [42,43,44]. There is a lot of evidence that has proved that auxin signal transduction, polar transport, and regulation are closely related to leaf morphology and development. The pin1 mutant, with inactivated *PIN1* (encoding a polar auxin transport protein) in *Arabidopsis*, showed wider leaves, fused cotyledon, and abnormal phyllotaxis, and the rosette leaves of axr mutant showed irregularity in shape and tended to curl downward [35,45]. Hay found that the accumulation of auxin can inhibit KNOX genes with *AS1* in the process of leaf primordium formation [31,45], and the auxin response factor ARF3/4 was indirectly repressed by the AS1-AS2 complex through activating the miR390-tasirR-ARF pathway [46]. This evidence allowed us to assumed that the BcAS2-BcAS1-1/2 complex was involved in the process of auxin affecting leaf morphological development in pak choi. Nevertheless, the correctness of our conjecture remains to be further studied in future.

## 5. Conclusions

In summary, we firstly cloned *BcAS2* from pak choi, which has a highly conserved LOB domain, and a high expression level in leaves. This study provided preliminary proof about the function of the BcAS2-BcAS1-1/2 complex in leaf polarity according to the yeast two-hybrid, co-localization, and transgenic assays. This finding contributed to a better understanding about the regulatory mechanism of leaf shape in pak choi or other Brassica species. In addition, the special leaf phenotype caused by overexpression of *BcAS2* can provide an insight for the morphological improvement of pak choi in order to increase its economic value.

## Data Availability

The Pak-choi cultivar “NHCC001”, *Nicotiana benthamiana* and *Arabidopsis thaliana* wild type used in this study were kindly provided by Professor Xilin Hou (Nanjing Agricultural University, Nanjing, China).

## Supplementary Materials

The following are available online at https://www.mdpi.com/2073-4425/12/1/102/s1, Figure S1: The prediction of interaction protein with BcAS2, Figure S2: The multiple sequence analysis between the potential interaction genes of *BcAS2* and the leaf polarity genes in *Arbidopsis thaliana* Table S1: Primers were used in this study, Table S2: Protein sequences of AS2 like genes in various plants.
