# Supplementary material for: Overexpression of a Pak Choi Gene, BcAS2, Causes Leaf Curvature in Arabidopsis thaliana
Source: Genes (Basel). 2021 Jan 15;12(1):102. doi: 10.3390/genes12010102 (PMC7830005; doi:10.3390/genes12010102)
Supplement: Supplementary file 1 [file genes-12-00102-s001.zip › Supplementary Figures.docx]

**
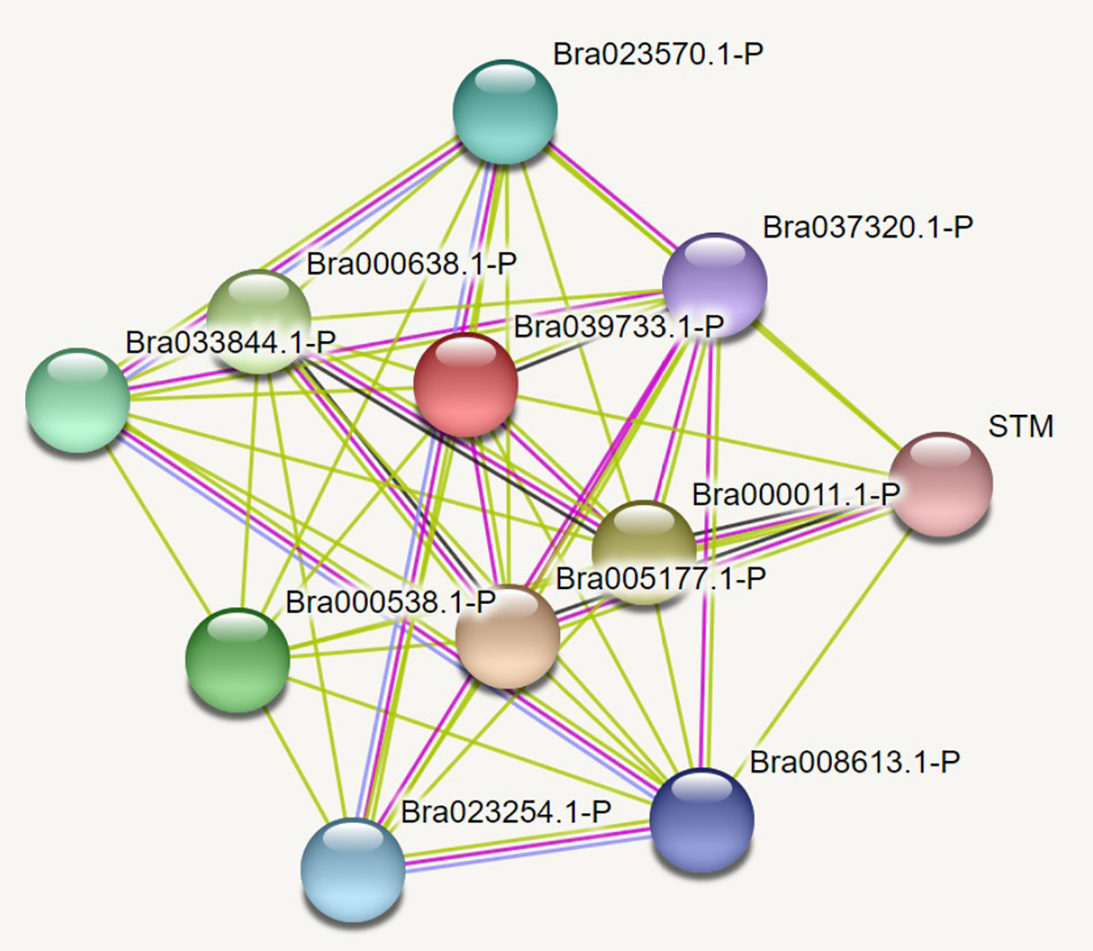
**

**Figure S1.** The prediction of interaction protein with BcAS2. The target proteins interacting with BcAS2 were predicted by STRING software. BcAS2, BcAS1-1, and BcAS1-2 corresponding to Bra039733, Bra000011, and Bra005177 respectively.


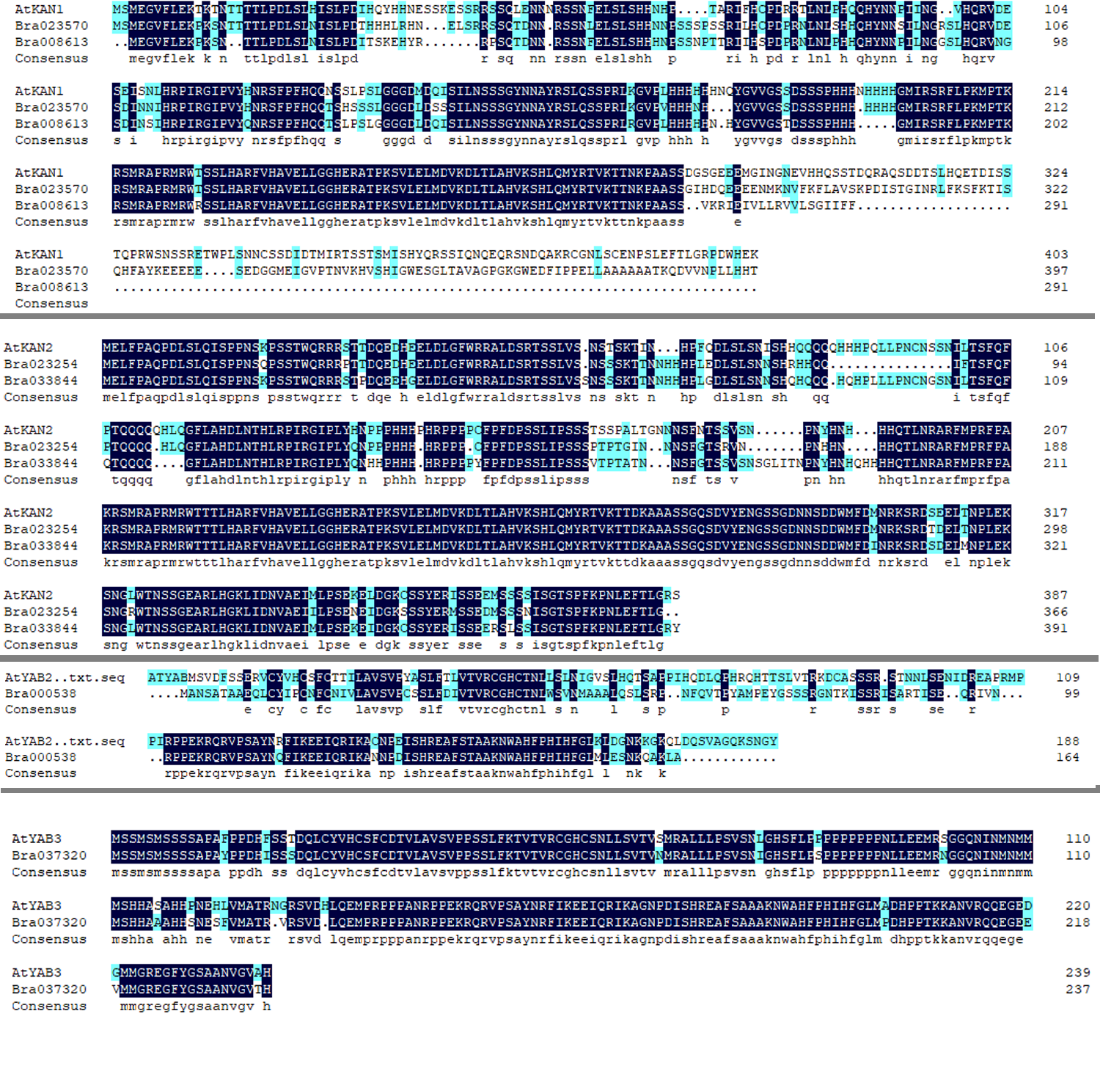


**Figure S2.** The multiple sequence analysis between the potential interaction genes of *BcAS2* and the leaf polarity genes in *Arbidopsis thaliana.* Gray lines are used to divide different groups, The homology from top to bottom is 63.74%，89.03%，50.01% and 92.50% respectively.
