# Supplementary figures and images for: Overexpression of a Pak Choi Gene, BcAS2, Causes Leaf Curvature in Arabidopsis thaliana
Source: Genes (Basel). 2021 Jan 15;12(1):102. doi: 10.3390/genes12010102 (PMC7830005; doi:10.3390/genes12010102)

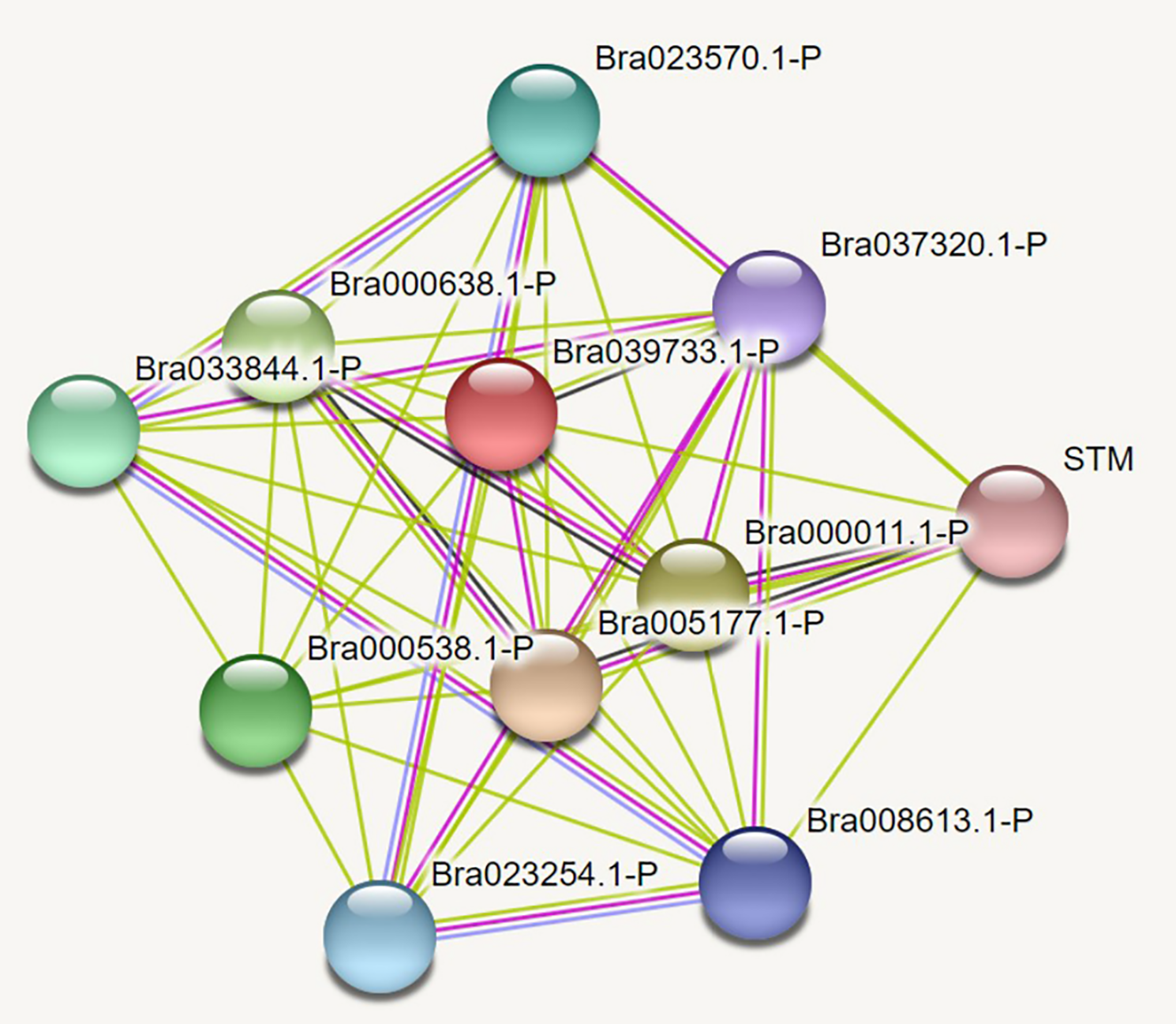

Supplement: Supplementary file 1 [file genes-12-00102-s001.zip › Figure S1.tif]

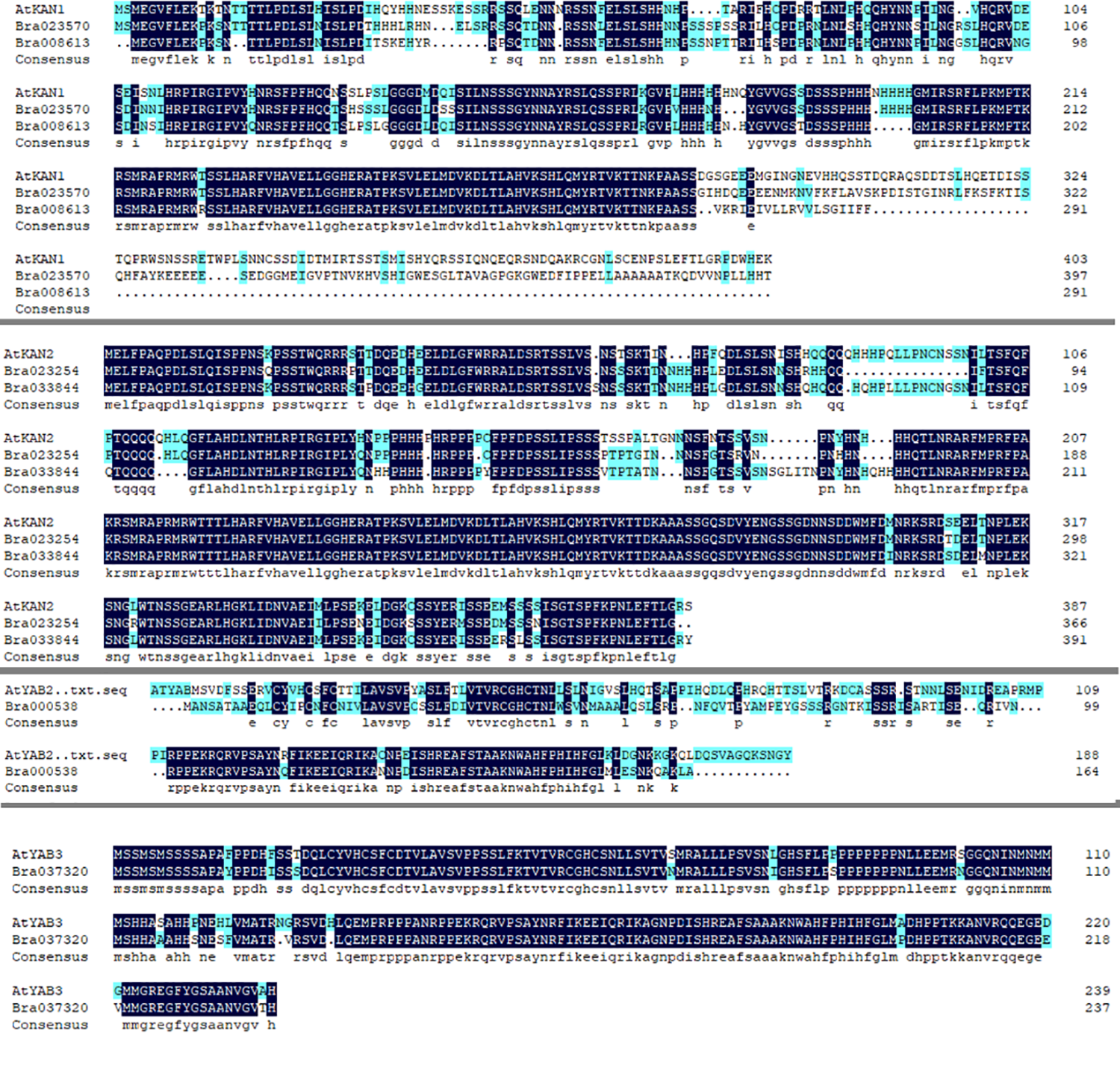

Supplement: Supplementary file 1 [file genes-12-00102-s001.zip › Figure S2.tif]
